# Redesigning TOR Kinase to Explore the Structural Basis for TORC1 and TORC2 Assembly

**DOI:** 10.3390/biom8020036

**Published:** 2018-06-01

**Authors:** Andrew Hill, Brad Niles, Andrew Cuyegkeng, Ted Powers

**Affiliations:** Department of Molecular and Cellular Biology, College of Biological Sciences, University of California Davis, Davis, CA 95616, USA; awhill@ucdavis.edu (A.H.); bjniles@ucdavis.edu (B.N.); ajcuyegkeng@ucdavis.edu (A.C.)

**Keywords:** HEAT repeats, mTOR pathway, rapamycin, AGC kinases

## Abstract

TOR is a serine/threonine protein kinase that assembles into distinct TOR Complexes 1 and 2 (TORC1 or TORC2) to regulate cell growth. In mammalian cells, a single mTOR incorporates stably into mTORC1 and mTORC2. By contrast, in *Saccharomyces cerevisiae*, two highly similar Tor1 and Tor2 proteins exist, where Tor1 assembles exclusively into TORC1 and Tor2 assembles preferentially into TORC2. To gain insight into TOR complex assembly, we used this bifurcation in yeast to identify structural elements within Tor1 and Tor2 that govern their complex specificity. We have identified a concise region of ~500 amino acids within the N-terminus of Tor2, which we term the Major Assembly Specificity (MAS) domain, that is sufficient to confer significant TORC2 activity when placed into an otherwise Tor1 protein. Consistently, introduction of the corresponding MAS domain from Tor1 into an otherwise Tor2 is sufficient to confer stable association with TORC1-specific components. Remarkably, much like mTOR, this latter chimera also retains stable interactions with TORC2 components, indicating that determinants throughout Tor1/Tor2 contribute to complex specificity. Our findings are in excellent agreement with recent ultrastructural studies of TORC1 and TORC2, where the MAS domain is involved in quaternary interactions important for complex formation and/or stability.

## 1. Introduction

Signaling networks allow cells to respond appropriately to changes in environmental and intracellular conditions, including nutrient availability, metabolic status, the presence of hormones and second messengers, as well as stress. Dysfunction within a network can have deleterious consequences for cells, including contributing to cancer in humans. Most networks include as principal components protein kinases and phosphatases, where co-localization within multi-protein complexes, often involving protein adaptors or scaffolds, can play an important regulatory role, both by ensuring proper interactions with substrates while preventing improper competing interactions [1,2]. Scaffold proteins often lack enzymatic activity, with a classic example being Ste5 in budding yeast, which organizes components of the mitogen-activated protein kinase (MAPK) cascade involved in mating and pseudo-filamentous growth [3,4]. Alternatively, scaffolds may possess enzymatic activity, for example the Pbs2 kinase that assembles with a distinct set of MAPK signaling components for osmoregulation in yeast [3]. Determining how signaling complexes are assembled and regulated, including the specific contribution of scaffolds, is essential for a complete understanding for the basis of specificity in cell signaling [2,4,5].

One important signaling network that regulates cell growth in eukaryotic organisms is defined by its central component, the rapamycin-sensitive TOR kinase (mTOR in mammalian cells) [6,7,8,9]. TOR is a large (~280 kDa) Ser/Thr protein kinase and a member of the atypical phosphatidylinositol 3-kinase-like kinase (PIKK) family of protein kinases [6,10]. TOR assembles with a number of other proteins to form two distinct protein complexes, termed TORC1 and TORC2 (or mTORC1 and mTORC2 in mammalian cells), where TORC1 is uniquely inhibited by rapamycin [7,11]. These complexes control diverse downstream processes, including protein synthesis, nutrient regulated gene expression, lipid biosynthesis, intracellular trafficking, and actin cytoskeletal organization, and thus collaboratively regulate multiple aspects of cell growth [6,7,9]. The activity of these complexes is responsive to nutrients, in particular amino acid availability, as well as cellular energy status and other regulatory inputs, including growth factors and insulin in mammalian cells [6,8,12]. In most eukaryotes, a single TOR protein is capable of assembling into TORC1 or TORC2, demonstrating all necessary sequence information required for interactions with binding partners from both complexes is contained within a single polypeptide chain [6,13,14,15]. 

By contrast, in budding yeast, *Saccharomyces cerevisiae*, two TOR paralogs exist, Tor1 and Tor2, the result of whole-genome duplication in the *Saccharomyces* lineage [13,16]. Sequence divergence of Tor1 and Tor2, which are approximately 65–70% identical, has resulted in the fact that while both kinases can function within TORC1, only Tor2 is capable of assembling into TORC2 [6,11,16]. The importance of Tor2 for both TORC1 and TORC2 activity was suggested by early genetic studies [17]. In particular, it was demonstrated that Tor2 has both a rapamycin-sensitive function (i.e., TORC1) that is shared with Tor1, as well as a Tor2-unique function (i.e., TORC2) that is not sensitive to rapamycin and is required for normal actin cytoskeleton dynamics and polarized cell growth [6,17]. As such, *TOR2* is essential but *TOR1* is not [18,19,20]. Interestingly, while Tor2 functions as part of TORC1, most if not all of TORC1 isolated from cells contains Tor1, indicative of preferential and/or stable association of Tor2 with TORC2-specific components [11,16,21]. Accordingly, specific sequences within Tor1 and Tor2 must govern their preferred assembly into TORC1 versus TORC2, thus defining these proteins as distinct kinase scaffolds.

TOR consists of multiple identifiable structural domains, where the N-terminal region is composed of arrays of helical repeats that fold into higher order solenoid-like elements that have been proposed to provide docking surfaces for specific binding partners [6,22]. The first portion of this array is composed of HEAT (Huntingtin, EF3, PP2A regulatory subunit A, and TOR) repeats, which are followed by FAT (FRAP, ATM, and TRRAP) or TPR (Tetratricopepetide repeat) repeats [6,10,22,23]. In general, each repeat is composed of two short α-helices linked by a short unstructured loop, such that the total length of each repeat averages about 40 amino acids [22,23,24]. Each repeat is flanked by spacer sequences that also vary in length and thus provide additional structural diversity to the overall conformation of the N-terminus of TOR. The presence of these helical repeats is a conserved feature of all of the PIKK kinases and in TOR is followed by the FRB (FKBP rapamycin binding) and kinase domains, where in TORC1 the FRB domain is targeted by the rapamycin-FKBP complex [6]. Accordingly, mutations within the FRB domain were isolated that confer dominant resistance to rapamycin, and thus enabled discovery of *TOR1* and *TOR2* in yeast [18,19,25,26]. Originally, approximately 20 HEAT repeats were identified in TOR by structure prediction and sequence analysis [22]. More detailed sequence alignment and bioinformatics analyses extended this number to a predicted 48 combined HEAT and FAT/TPR repeats [23,27]. To date, available data from both low as well as higher resolution structural studies of the TOR complexes are largely consistent with an extensive α-helical domain topology within TOR [28,29,30,31,32,33,34]. However, these studies have revealed a more varied architecture within the N-terminus of TOR, which has been subdivided into distinct structural regions termed Spiral (or, alternatively, Horn), Bridge, and CAP domains, followed by the FAT and kinase domains [29,32]. At the extreme C-terminus of TOR resides what has been termed the FATC domain [10].

In yeast, TORC1-specific binding partners for Tor1/Tor2 include Kog1 and Tco89, whereas TORC2-specific partners include Avo1–Avo3 and Bit61; the protein Lst8 is a common partner for both complexes [11,16,21]. In mammalian cells, mTORC1 contains Raptor, the homolog of Kog1, and mTORC2 contains both Rictor and hSin1, homologs of Avo3 and Avo1, respectively; the Lst8 homolog, mLst8/GβL, is also known to interact with both mTORC1 and mTORC2 [14,15,35]. Previous biochemical studies of yeast and mammalian complexes have demonstrated that these complex-specific partners are likely to interact with the N-terminal region of TOR, whereas Lst8/mLst8 interacts with the kinase domain and proximal sequences. In many cases, these assignments have been confirmed and extended by recent ultrastructural studies of TORC1 and TORC2. Moreover, as first demonstrated for yeast using biochemical approaches [36], these complexes function as dimers, a finding that has also been confirmed by recent ultrastructural studies. 

An outstanding question that remains is the identity of structural differences between Tor1 and Tor2 that account for the scope of functional activities associated with each protein, as well as the unique assembly of Tor2 into TORC2. Early studies demonstrated that the N-terminal region of Tor2 is responsible for its Tor2-unique function and, presumably, assembly into TORC2 [19,37]. Specifically, a chimeric TOR protein composed of the entire N-terminal HEAT repeat-containing domain and most of the FAT domain fused to the remaining C-terminal portion of Tor1, is capable of providing complete Tor2 activity when expressed in a strain lacking an endogenous *TOR2* gene [19,37]. The precise identity of the segment(s) within the N-terminal region that confers this specificity has not been determined. Similarly, the identity of sequences within Tor1 that underlie its obligate assembly into TORC1 have not been identified. Here, we describe experiments that address these issues and identify a concise domain within the proximal N-terminal region of Tor1 and Tor2 that is critical for their assembly into TORC1 versus TORC2. By comparing our results to recent structural studies of the mammalian and yeast TOR complexes, we have been able to gain additional insights into the architectural role of specific helical domains within TOR during formation of these complexes.

## 2. Results and Discussion

### 2.1. Experimental Approach

We sought to identify specific amino acid sequences within Tor2 that determine its essential Tor2-unique function as well as guide assembly into TORC2. Accordingly, we used a plasmid-based system to express chimeric *TOR* genes that possessed different regions of TOR2 embedded within an otherwise *TOR1* gene. The starting plasmid used was pPL130, which carries a full-length rapamycin-resistant *TOR1-1* gene, under control of its endogenous promoter, fused to the three copies of the HA-epitope at its N-terminus (see Materials and Methods). We used recombinant DNA methods to replace defined *TOR1* sequences with corresponding *TOR2* sequences within this plasmid. Because a prior study observed that sequences N-terminal to the FRB and kinase domains confer Tor2 specificity [19], we limited our analysis to this region of Tor2. To guide the interpretation of our results and to maximize the likelihood that chimeras reconstituted at least normal Tor1 function, we designed junctions between Tor1 and Tor2 sequences (termed “switch points”, see Supplemental Table S1) according to predicted positions of HEAT and FAT/TPR repeats, based on prior structure prediction and sequence alignments of the TOR gene family [23,27]. While it was clear at the outset that some of these assigned α-helical repeats and associated linker regions would likely require revision following structural studies of the TOR proteins, they represented a good first approximation for use in chimera design for this study.

**Figure 1:**
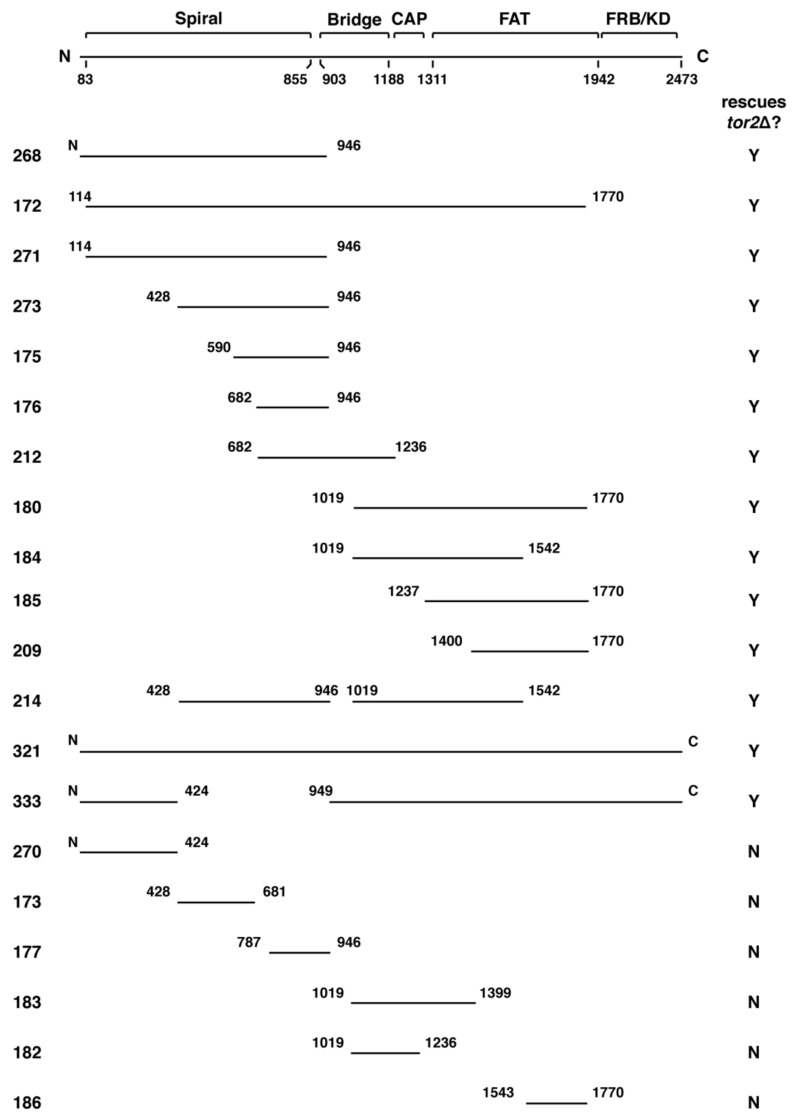
Summary of chimeric *TOR1*–*TOR2* genes constructed for this study. The diagram at the top depicts the domain architecture of the Tor protein. Below are schematics of plasmids that express different regions of *TOR2* imbedded in *TOR1*. Numbers on either end of the black bars denote amino acid positions of the Tor2 protein and “N” and “C” refer to the N- and C-termini, respectively. Numbers on the far left correspond the plasmid number for each chimera (listed in Tables 1 and 3). The far-right column indicates whether a given chimera rescued the lethality of a *tor2*Δ strain (Y = Yes, N = No). FAT: FRAP, ATM, and TRRAP; FRB: FKBP rapamycin binding.

Plasmids expressing chimeras were introduced into WT as well as *tor1*Δ cells and tested for *TOR1* function by examining their ability to confer resistance to rapamycin. As noted below, a few chimeras we constructed did not carry the *TOR1-1* allele. Therefore, these constructs were tested for *TOR1* function instead by examining their ability to allow grow of *tor1*Δ cells at 37 °C, taking advantage of the observation that loss of *TOR1* renders cells temperature sensitive in the W303 strain background [21]. Only chimeras that provided rapamycin resistance or temperature-resistant growth in *tor1*Δ cells were examined further. To test for *TOR2* function, plasmids were introduced into heterozygous *TOR2*/*tor2*Δ diploid cells, followed by sporulation and tetrad dissection. Successful isolation of plasmid-containing *tor2*Δ haploid cells was taken as evidence that a given chimera provided Tor2-specific activity. For comparison, plasmids expressing WT *TOR1* (pPL132) or *TOR2* (pPL089) were tested in parallel. As an additional control, we constructed a plasmid (pPL172) that expressed all of the HEAT repeats in the Spiral domain as well as most of the FAT domain of *TOR2* within the context of an otherwise *TOR1* gene. This latter construct is essentially identical to the original *TOR2*–*TOR1* hybrid described previously [19]. For simplicity, throughout the text we refer to each chimera by its designated plasmid number (Figure 1). 

### 2.2. Genetic Analyses of TOR2–TOR1 Chimeras

Using the approach outlined above, we identified approximately twenty distinct *TOR2–TOR1* chimeras that provided Tor1 activity and, accordingly, functioned within TORC1 (Figure 1 and Table 1). Remarkably, many of these chimeras were also capable of rescuing the lethality of *tor2*Δ cells, demonstrating that multiple distinct sequences within Tor2 provide sufficient Tor2 activity within the context of TORC2 (Figure 1 and Table 1). Inspection of Figure 1 reveals that several unique regions throughout Tor2 rescued the lethality of a *tor2*Δ mutant (e.g., compare chimeras 273 and 185). These findings suggest that Tor2 identity is not determined by a single discrete structural element within Tor2. Chimeras that were unable to function as Tor2 possessed, in general, smaller portions of Tor2 and/or switch points that interrupted larger chimeras that functioned as Tor2. Together these findings indicate that while both the size and position of Tor2 sequences are important for Tor2 activity, multiple independent regions satisfy these requirements.

**Figure 2:**
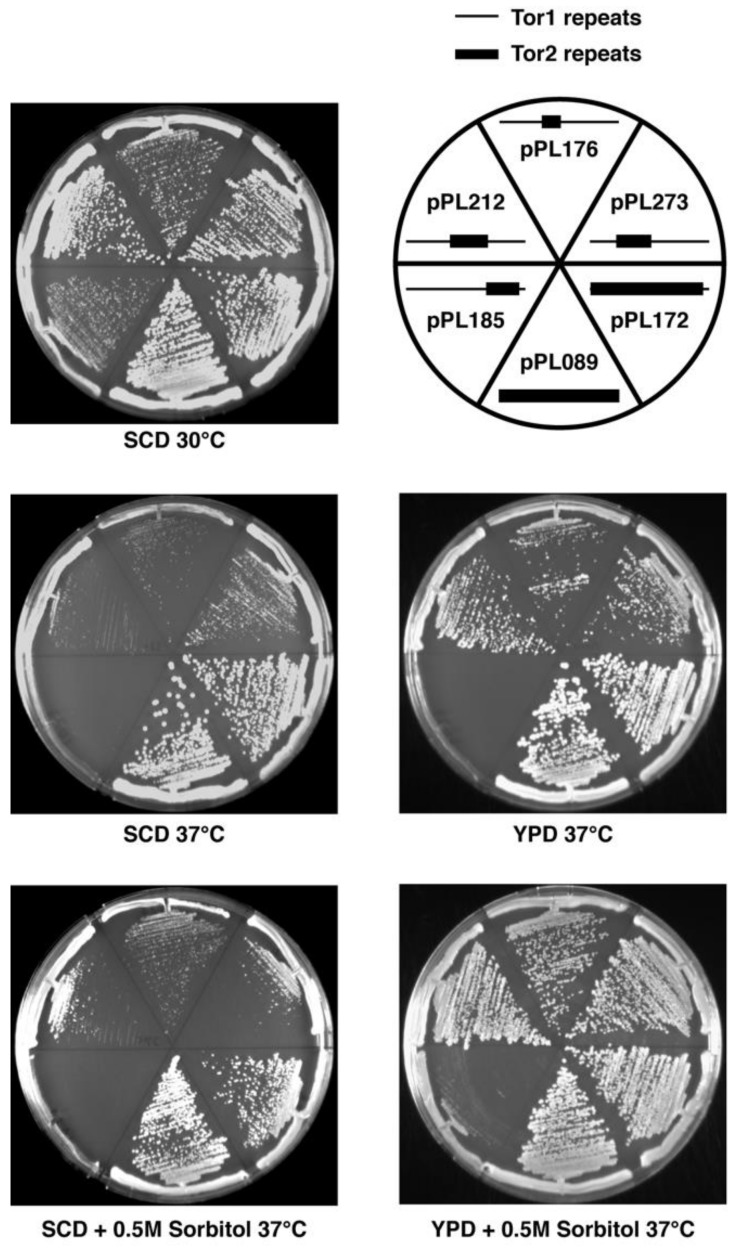
Phenotypes of selected chimeras in *tor2*Δ cells on solid medium. Plates contained the indicated media and were grown for 2 days at the indicated temperatures and then photographed. Legend depicts chimeras tested on the corresponding plates. Schematic indicates N-terminal Tor2 (heavy lines) versus Tor1 (thin lines) regions in each chimera.

While the chimeras described above suppressed the lethality of *tor2*Δ cells, we observed that most conferred growth defects, particularly at elevated temperatures. In general, chimeras possessing Tor2 sequences that corresponded to more C-terminal regions and/or possessed smaller sections of Tor2 displayed temperature sensitive growth (Figure 2, Table 1). For example, chimera 185 (CAP-FAT domains) displayed weaker growth compared to chimera 273 (Spiral-Bridge domain) under all conditions tested, but these differences were particularly evident at 37 °C. We observed that all chimeras that possessed discrete regions of Tor2 displayed some degree of temperature sensitivity, compared to chimera 172, which contains the majority of Tor2 (Figure 2, Table 1). Thus, we conclude that robust Tor2 function requires extended Tor2 sequences throughout its N-terminus.

**Figure 3:**
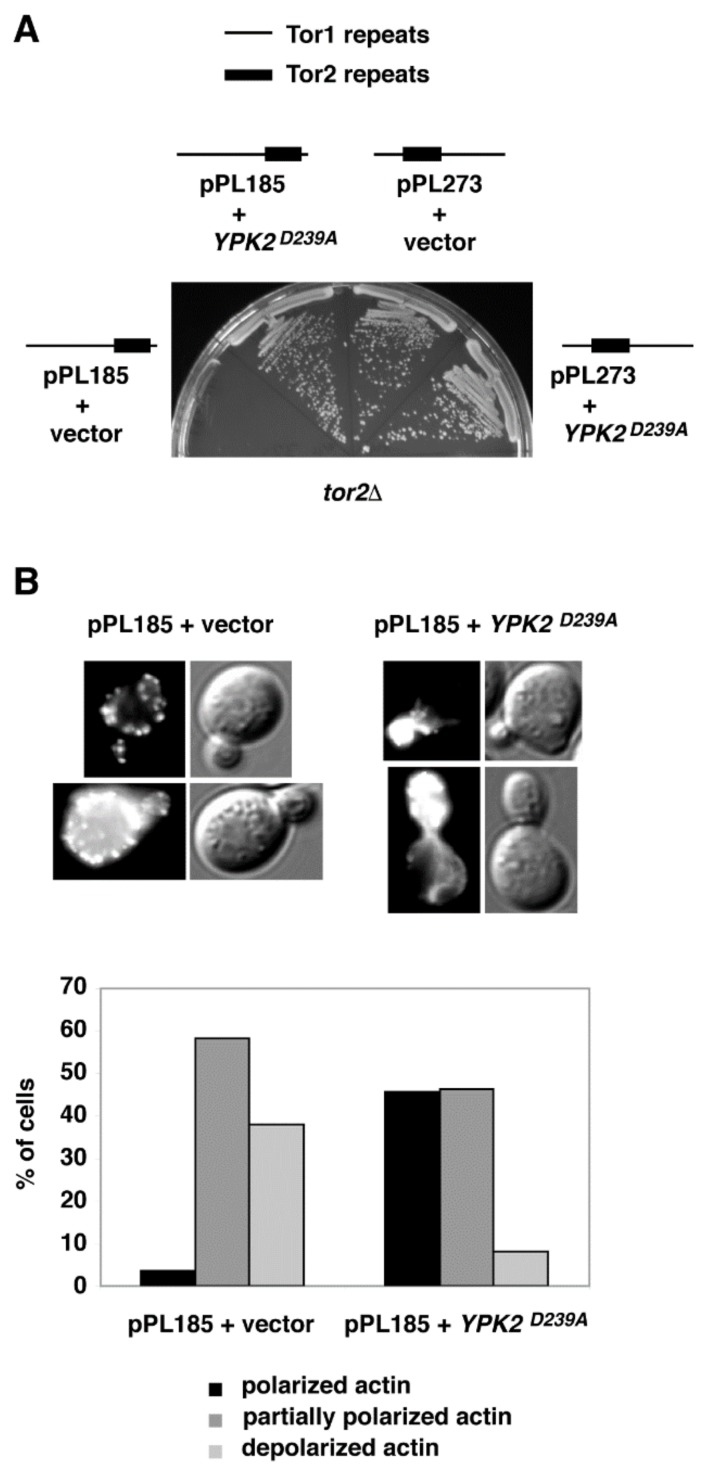
Chimera 185 possesses TORC2-specific defects. (**A**) Rescue of impaired growth at 37 °C of chimera 185 in *tor2*Δ cells by expression of *YPK2^D239A^* on SCD plates containing 0.5 M sorbitol. (**B**) Rescue of actin polarization defects of chimera 185 *tor2*Δ cells at 37 °C by expression of *YPK2^D239A^*.

For several chimeras, growth at elevated temperatures was improved by inclusion of rich media and/or the osmotic stabilizer sorbitol (Figure 2, Table 1). Because rescue of temperature-sensitive growth by sorbitol is a hallmark of a Tor2-unique defect [17], we tested this directly for chimera 185. Specifically, we introduced into *tor2*Δ cells expressing this chimera an allele of the AGC kinase Ypk2 (*YPK2^D239A^*) that bypasses a requirement for TORC2 activity for cell viability [38]. We observed that the extreme growth defect of this chimera was rescued by expression of *YPK2^D239A^* (Figure 3A). By contrast, no significant change in growth of *tor2*Δ cells carrying chimera 273 was observed in the presence of *YPK2^D239A^*, consistent with its more robust behavior. In addition, we observed that actin polarization defects associated with chimera 185, another distinct hallmark of a Tor2-deficiency [39], were also rescued by expression of *YPK2^D239A^* (Figure 3B). Taken together, these results confirm the importance of N-terminal sequences for Tor2-specific activity, yet also underscore an important secondary role of C-terminal sequences within the CAP and FAT domains.

### 2.3. Monitoring Assembly of Chimeras into TORC1 and TORC2

**Figure 4:**
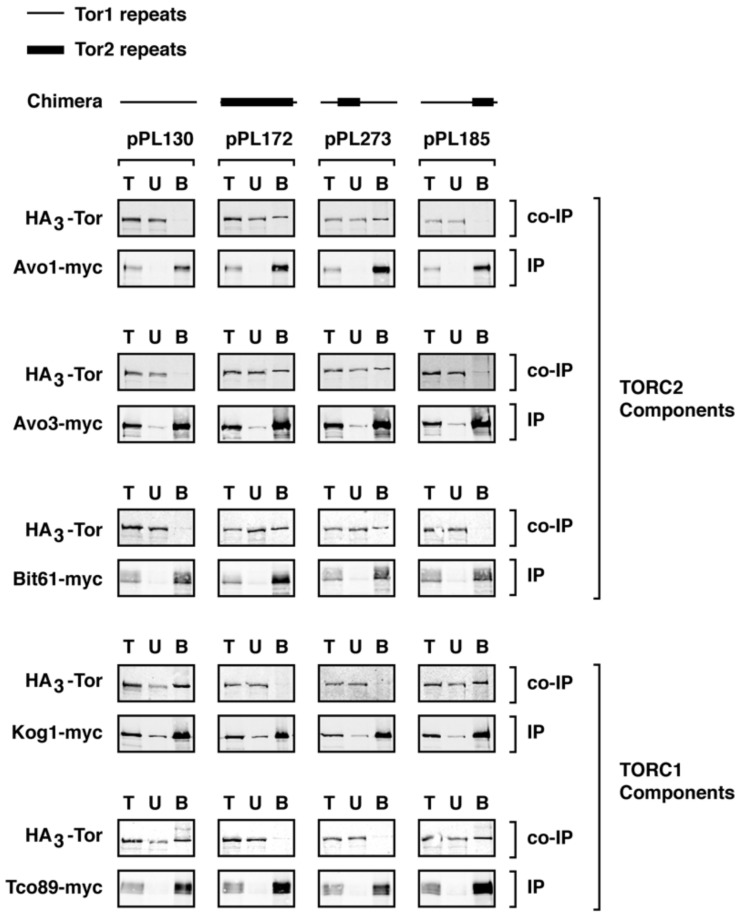
Assembly of chimeras into specific TOR Complexes. Indicated chimeras were assayed for assembly into TORC1 and TORC2 by examining co-immunoprecipitation between specific myc-tagged components (IP) and HA3-tagged chimeric Tor proteins (Co-IP). T = total material prior to IP; U = unbound material; B = bound material that indicates association between components.

We next tested whether phenotypes associated with specific chimeras correlated with their assembly into TORC1 and/or TORC2. We focused on two chimeras, 273 and 185, that displayed distinct phenotypes with respect to *TOR2*-like behavior in *tor2*Δ cells, as described above. Constructs were introduced into cells that expressed functional myc-epitope tagged versions of endogenous components of TORC1 or TORC2 together with HA3-tagged chimeras [21]. Extracts were prepared and co-immunoprecipitation experiments were conducted to examine association between each myc-tagged component and a given chimera (see Material and Methods). As demonstrated previously, this approach provided a qualitative assay for stable interactions between Tor1/Tor2 and their binding partners [11,16,21]. For comparison, in parallel we examined pPL130 (expressing *TOR1*) as a TORC1-specific control, as well as chimera 172, which, as expected, assembled exclusively into TORC2 (Figure 4).

We observed that chimera 273 assembled stably into TORC2, based on co-immunoprecipation with Avo1-myc, Avo3-myc, and Bit61-myc (Figure 4). By contrast, no significant association was observed between this chimera and Kog1-myc or Tco89-myc, demonstrating that, like full length Tor2 [11,16,21] and chimera 172, chimera 273 does not form stable interactions with TORC1 components (Figure 4). Importantly, this result demonstrates that a minimal region within the N-terminus of Tor2 was sufficient to direct an otherwise Tor1 protein stably into TORC2. By contrast, chimera 185 formed stable interactions with TORC1 but not with TORC2 partners, demonstrating that these more C-terminal sequences in Tor2 were insufficient to re-direct the stable assembly of this chimera from TORC1 into TORC2 (Figure 4). This finding is consistent with the weaker Tor2-specific phenotypes afforded by chimera 185. Interestingly, these biochemical properties of chimera 185 are the inverse of Tor2, which can function within TORC1 but forms stable associations with TORC2 components. Thus, our findings illustrate that it is possible for a Tor protein to associate stably with one complex but provide sufficient functional activity within both complexes to support cell viability. 

**Figure 5:**
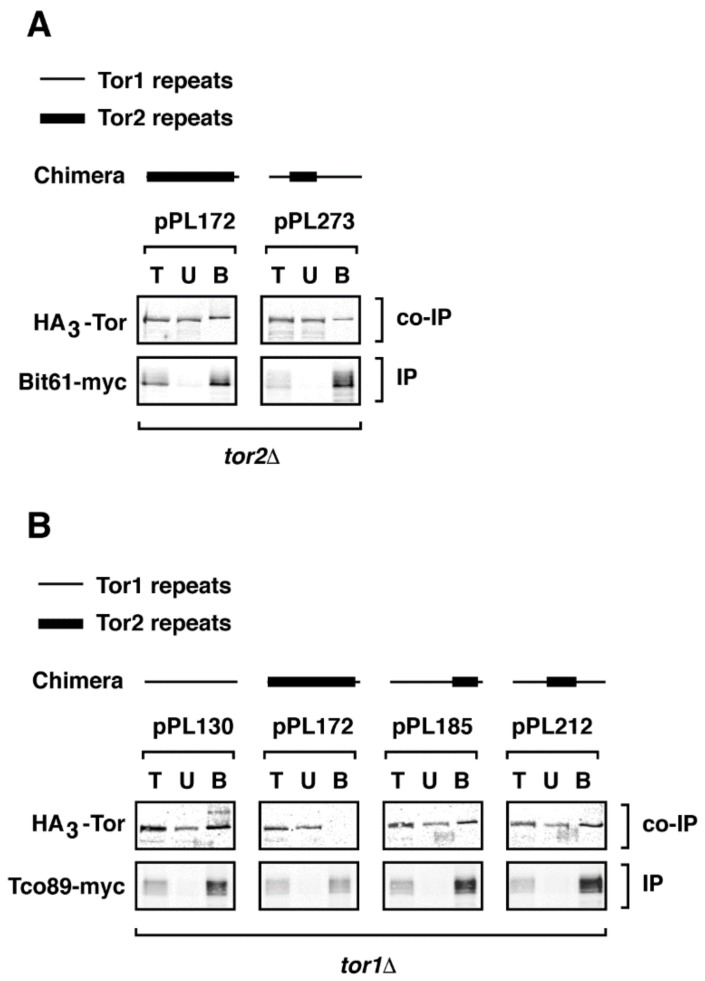
Complex specificity of chimeras does not require expression of endogenous *TOR1* or *TOR2*. Indicated chimeras were expressed in (**A**) *tor1*Δ and (**B**) *tor2*Δ strains and co-immunoprecipitation experiments were performed as described in the legend to Figure 4.

Because TORC1 and TORC2 function as homodimers or higher order oligomers [36,40], we wanted to test whether interactions we observed between chimeras and complex-specific binding partners were mediated by endogenous Tor1 or Tor2 proteins. Specifically, we wished to determine whether chimera 273 required endogenous Tor2 to assemble into TORC2 and whether chimera 185 required endogenous Tor1 to assemble into TORC1. Accordingly, we performed co-immunoprecipitation experiments in strains carrying chromosomal deletions of *TOR1* or *TOR2*. For this study, we restricted our analyses to the non-essential TORC1 and TORC2 components, Tco89-myc and Bit61-myc, respectively, because of impaired growth rates when epitope tagged versions of essential subunits were combined with *TOR1* and *TOR2* deletions. We observed that chimera 185 co-immunoprecipitated with Tco89-myc in a *tor1*Δ strain and that chimera 273 co-immunoprecipitated with Bit61-myc in *tor2*Δ strain (Figure 5A,B). Based on these results, we conclude that endogenous Tor1 or Tor2 proteins are not required for chimeras to assemble into specific complexes. These findings also demonstrate that despite the absence of Tor1 in *tor1*Δ cells, Tor2 (in the form of chimera 172) is not stably incorporated into TORC1, at least under these experimental conditions (Figure 5B).

**Figure 6:**
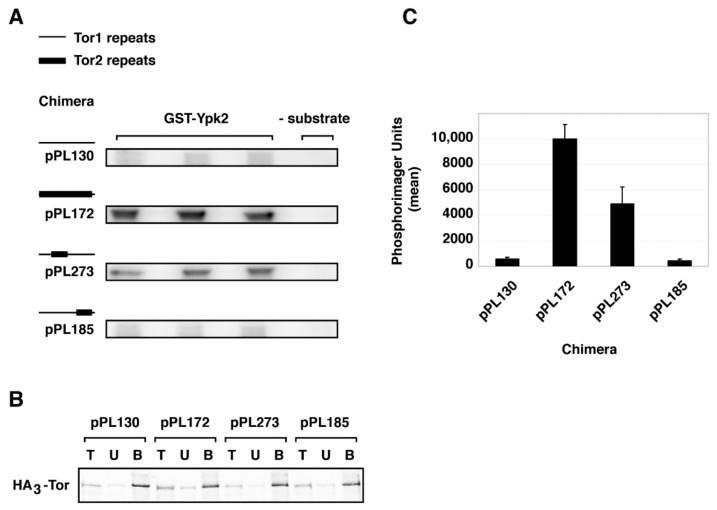
Recognition of TORC2-specific substrate Ypk2 by chimera 273. (**A**) Autoradiograph of triplicates of in vitro kinase reactions using immuno-purified complexes containing indicated chimeras and recombinant GST-Ypk2. (**B**) Western blot of loading controls demonstrating that essentially equivalent amount of TOR chimeras were present in the immunoprecipitated complexes (bound (**B**) fractions) used for kinase reactions. (**C**) Quantification of data in (**A**), where total phosphorimager units are indicated. Data are presented as means +/− standard deviation (SD) of the triplicate samples shown in (**A**) and were normalized for levels of Tor protein present.

To test whether stable assembly of chimera 273 into TORC2 correlated with substrate-specific kinase activity, we tested the ability of the chimeras analyzed above to phosphorylate Ypk2, a TORC2-specific substrate. Here we immunoprecipitated chimeras using the HA3-epitope and examined their ability to phosphorylate recombinant Ypk2, in vitro. We chose this substrate as we have demonstrated previously that there is no cross-recognition by TORC1, by contrast to the paralog Ypk1, where there is weak recognition by TORC1 [41]. We observed that chimera 273 displayed significant phosphorylation of Ypk2, although not as robust as the activity afforded by the control chimera 172 (Figure 6). By contrast, no significant phosphorylation of Ypk2 was observed by chimera 185, which was similar to what was observed with Tor1 (pPL130) (Figure 6).

### 2.4. A Major Assembly SpecificityDomain in TOR

Our above findings reveal that a relatively concise region of ~500 amino acids of Tor2 in chimera 273, corresponding to a majority of the Spiral and a portion of the Bridge domains, are sufficient to drive the stable assembly of TOR into TORC2. By contrast, a more C-terminal segment containing the CAP and FAT domains in chimera 185 do not on their own confer stable TORC2 assembly but instead retains specificity for TORC1. Similarly, chimera 212, which includes the C-terminal portion of the Spiral domain and all of the Bridge domain, and is therefore partially overlapping with chimera 273, also remains stably associated with TORC1 (Figure 5A). Finally, our attempts to define a more minimal region within chimera 273 have so far been unsuccessful; for example, chimeras 173 and 177, each containing about 250 amino acids of Tor2 that correspond to a smaller section of chimera 273, can function as Tor1 but neither confers Tor2-specific activity (Figure 1 and Table 1). We conclude that sequences in Tor2 located within chimera 273 are particularly important for TORC2 assembly and therefore refer to this segment as a major assembly specificity (MAS) domain. This conclusion is consistent with data reported previously that Tor2-specific activity requires sequences within the N-terminal one-third of this protein [37].

**Figure 7:**
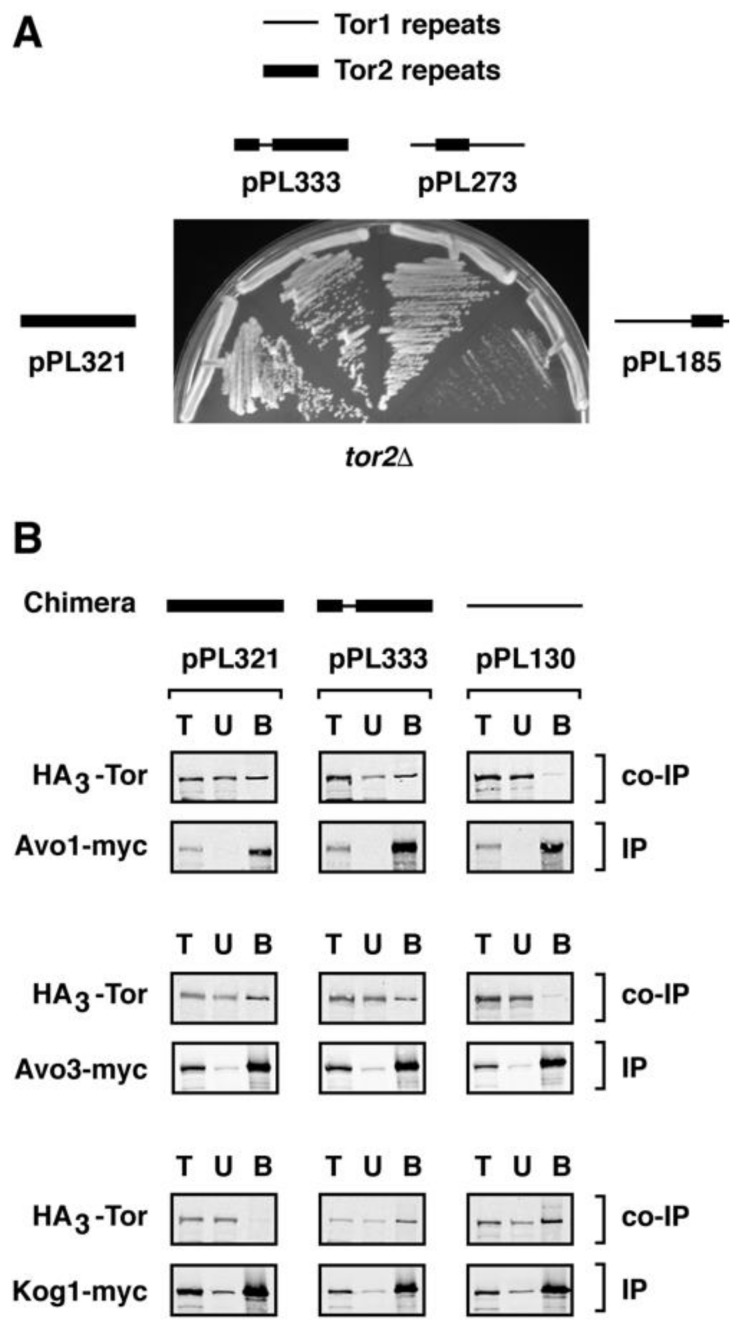
Testing the effects of replacing the Major Assembly Specificity (MAS) domain from Tor2 with Tor1 in chimera 333. (**A**) Growth properties of chimera 333 at 30 °C, demonstrating robust rescue of the lethality of *tor2*Δ cells. (**B**) Chimera 333 associates stably with components of both TORC1 and TORC2.

To test the generality of these results, we asked whether a reciprocal situation might exist, namely, if the corresponding region of Tor1 would be capable of facilitating stable incorporation of Tor2 into TORC1. Toward this end, we started with plasmid pPL321 that expresses the entire *TOR2* gene under control of its endogenous promoter. We replaced the Tor2 MAS domain with corresponding sequences from Tor1, to create chimera 333. We observed that this chimera rescued the lethality of a *tor2*Δ strain and, moreover, behaved essentially like full-length Tor2 (pPL321) under all conditions tested (Figure 7A and Table 1). In addition, both Tor2 and chimera 333 interacted with TORC2-specific components Avo1-myc and Avo3-myc in co-immunoprecipitation experiments (Figure 7B). Remarkably, however, chimera 333 also co-precipitated with Kog1-myc, demonstrating that this chimera also stably incorporates into TORC1 (Figure 7B). From these data we conclude that the MAS domains of both Tor1 and Tor2 are important for their preferential assembly into TORC1 versus TORC2, respectively.

**Figure 8:**
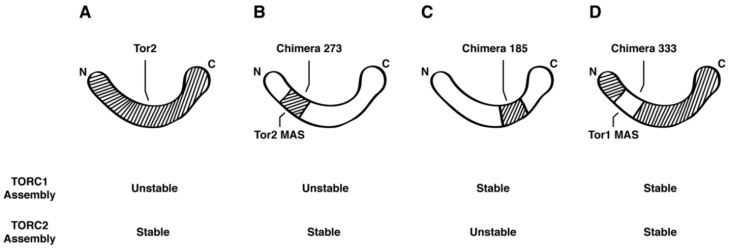
Model depicting the role of the Tor1 and Tor2 MAS domains in contributing to the complex specificity of TORC1 and TORC2 assembly. Stripped lines refer to Tor2 sequences, while the non-stripped diagram depicts Tor1 sequences.

We interpret our data regarding the assembly of Tor2 into TOR Complexes 1 and 2 in terms of the model presented in Figure 8. The Tor2 MAS domain is sufficient to convert an otherwise Tor1 protein into a chimera (i.e., 273) (Figure 8B that incorporates stably into TORC2. This protein is still capable of providing TORC1 activity but does not stably associate with TORC1 components. By contrast, multiple distinct regions of Tor2 outside of the MAS domain (e.g., chimera 185) (Figure 8C) are sufficient to confer adequate Tor2 activity to suppress the lethality of endogenous *TOR2*, but only confer weak/unstable association with TORC2 components. Finally, when the Tor1 MAS domain is incorporated into a chimera that is otherwise Tor2 (i.e., chimera 333) (Figure 8D), the MAS domain of Tor1 is sufficient to drive assembly with TORC1 components. However, because all of the flanking sequences are Tor2, we argue that these are sufficient to function together and facilitate stable interactions with TORC2 components. This situation is thus reminiscent of mTOR, which can stably incorporate into both mTORC1 and mTORC2. A corollary of this conclusion is that it may be possible to redesign mTOR to preferentially associate with a specific complex, for example by mutating residues within the corresponding MAS domain of mTOR. Such modified mTOR proteins may be useful in targeting changes in activity to specific complexes.

### 2.5. Toward Understanding Quaternary Interactions Important for TORC1 and TORC2 Assembly

**Figure 9:**
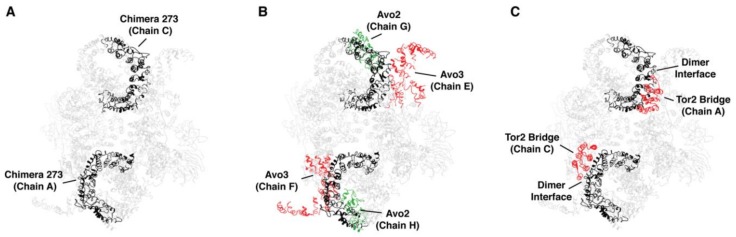
The Tor2 MAS domain of chimera 273 is involved in several quaternary interactions in TORC2. (**A**) Tor2 MAS (chimera 273) (black) mapped onto the cryo EM structure of TORC2 [30]. (**B**) Interactions between Tor2 MAS (black) and Avo2 (green) and Avo3 (red). (**C**) Interactions between Tor2 MAS (black) and Tor2 sequences (red) at the dimer interface. Protein chains are labeled according to nomenclature described in [30]. For clarity, the unstructured region between residues 869-914 in Tor2 in chimera 273 is not displayed.

Our findings demonstrate that specific structural elements in Tor1 and Tor2, particularly within what we have termed the MAS domain, contribute to stable incorporation of these proteins into specific TOR complexes. To gain insight into whether these regions participate in specific quaternary interactions, we examined our findings within the context of recently published structural models for TORC1 and TORC2. In particular, by mapping Tor2 sequences corresponding to chimera 273 onto the structure for yeast TORC2, determined by cryo-EM [30], we observed that these sequences correspond to the region of the spiral domain that occupies a prominent position along the outer surface of the TORC2 dimer (Figure 9A).

Using tools available in the program UCSF Chimera [42], we identified predicted atomic contacts between the Tor2 MAS within chimera 273 and other components of TORC2, including interactions involving both chains of Tor2 (Table 2). Because of the resolution of this structure, we understand these contacts represent at present only potential areas of interaction. We observed that these sequences form a majority of interactions between Tor2 and both Avo2 as well as Avo3 (Figure 9B and Table 2). Moreover, these sequences are also involved in interactions that form the dimer interface, where helices within the spiral domain of one chain of Tor2 interact with helices located within the bridge domain of the other Tor2 chain [30] (Figure 9C and Table 2). Importantly, chimera 273 is involved in all inter-Tor2 contacts located within the N-terminus of Tor2 (Table 2, compare to chimera 172). Importantly, in recently described structures of TORC1/mTORC1, this dimer interface is also involved in interactions with Raptor and, presumably, the yeast ortholog Kog1 [29,32]. Thus, our identification of the MAS domain is consistent with involvement of this region in interactions with both sets of complex-specific binding partners.

One question raised by these findings is the extent to which interactions between Tor2 chains within the TORC2 dimer versus interactions between Tor2 and Avo2-Avo3 help drive complex assembly and/or stability. In this regard, for chimera 273 the dimer interface necessarily consists of Tor2 sequences within the spiral domain and Tor1 sequences within the bridge domain. By contrast, chimera 212, which partially overlaps chimera 273, includes both segments of the N-terminal dimer interface (Figure 1, Table 2). Thus, for chimera 212, the dimer interface consists entirely of Tor2 sequences. However, we observed that this chimera remains stably associated with TORC1, suggesting that homotypic interactions at the dimer interface are insufficient to determine complex specificity. We note that compared to chimera 273, chimera 212 is missing all predicted interactions with Avo2 and has a reduced number of Avo3 contacts, suggesting these differences may play an important role in the preference of chimera 212 for TORC1 (Table 2). We remain cautious with this interpretation for at least two reasons. First, the precise identities and total number of interactions between Tor2 and its binding partners within TORC2 are presently unknown, given the resolution of published structures and the fact that Avo1-Avo3 are represented incompletely in the model used for this analysis [30]. Second, while we have identified regions of difference between Tor1 and Tor2 that account for functional and structural specificities, a high level of similarity remains between these proteins and it is presently unknown at the amino acid level what accounts for differences in complex assembly specificity.

We observed that chimera 333, which possesses the Tor1 MAS domain but is otherwise composed completely of Tor2 sequences, contains all predicted interactions between Tor2 and Avo1, in addition to a subset of interactions with both Avo2 and Avo3 (Table 2). This observation is consistent with our conclusion above that interactions outside of the MAS domain are important for complex formation. Moreover, these findings suggest a specific model for the ability of chimera 333 to assemble into both TORC1 and TORC2. Thus, we suggest that this Tor protein may interact initially either with Kog1 (via the Tor1 MAS domain) and be driven toward TORC1 formation or, alternatively, interact with Avo1 (via Tor2-specific sequences) and be driven toward TORC2 assembly. An interesting question posed by this model is whether a similar pathway exists in mammalian cells, where a single mTOR must associate in a competitive fashion with distinct binding partners to form mTORC1 versus mTORC2. Exploring further the assembly pathway of the yeast complexes using these chimeric Tor1-Tor2 proteins will be an invaluable tool to test these hypotheses, as well as identifying precise amino acid residues within the TOR proteins required for the assembly of each complex.

Finally, we note that several other eukaryotic species also possess more than one TOR gene, including *Schizosaccharomyces pombe*, which harbors two TOR orthologs [43], and Trypanosomes, which possess multiple *TOR* genes [13]. Importantly, at least for *S. pombe*, there is a demonstrated preference of assembly of Tor1 and Tor2 into distinct complexes [44]. Thus, exploring the basis for this specificity as well as studying differences between the *TOR* genes of other organisms may provide additional insight into the structural features that determine specific TOR complex assembly and function.

## 3. Materials and Methods

### 3.1. Strains, Media, General Methods

Yeast strains used in this study are listed in Table 3. Culture medium used was synthetic complete dextrose (SCD) (0.8% yeast nitrogen base without amino acids, pH 5.5, 2% dextrose) supplemented with amino acids as described [45]. Rapamycin (Sigma-Aldrich, St. Louis, MO, USA) was dissolved into dimethyl sulfoxide (DMSO) and added to a final concentration of 0.2 µg/mL. IPTG was dissolved in ddH_2_0 and added to a final concentration of 42.5 µM. Anti-hemagglutinin (HA) (12CA5) monoclonal antibody was purchased from Roche Diagnostics (Indianapolis, IN, USA). Anti-myc (9E10) monoclonal antibody was purchased from Covance (Princeton, NJ, USA). Yeast strains were transformed using the lithium acetate procedure [46]. Gene deletion strains were created by replacing an entire open reading frame using selectable markers, as described previously [21], except that the *TOR1* gene in PLY497 was replaced using the *TRP1* marker rather than *HIS3MX6*. Purification of recombinant GST-tagged Ypk1 was carried out as described [41].

### 3.2. Plasmid Construction

Plasmids used in this study are indicated in Table 4. Plasmid pPL130 was constructed in multiple steps. The starting plasmid was pYDF23, which carries the *TOR1-1* allele under control of its native promoter [20]. We introduced sequences corresponding to three copies of the HA epitope (HA3) at the N terminus of the coding region of *TOR1-1*, by PCR amplification using genomic DNA from strain PLY298 [21] that expresses *HA3-TOR1* to generate a linear fragment of DNA that encodes the promoter region of *TOR1* followed by the N-terminus of *TOR1* fused to HA3. This fragment was used in a co-transformation along with gapped pYDF23 to generate an intact HA3-tagged *TOR1-1* gene.

Subsequent *TOR1–TOR2* chimera plasmids were constructed using PCR-amplified regions of *TOR2* or *TOR1* that were combined using overlap extension (SOEing) [49]. Appropriate sequences were amplified and either used in subsequent reactions or were directly cloned by restriction digest and ligation using pPL130 and Rapid DNA Ligation kit (Sigma-Aldrich). The precise positions of junctions between *TOR1* and *TOR2* for each plasmid are listed in Supplementary Table S1.

### 3.3. Immunoaffinity Purification of TOR and TOR-Complex Binding Partners 

Co-immunoprecipitation experiments were conducted essentially as described [21]. Yeast strains expressing HA epitope-tagged TOR and myc epitope-tagged binding partners were grown at 30 °C to 0.5 OD_600_/mL in SCD minus leucine media. Cells were pelleted, washed in cold H_2_O, pelleted again, and resuspended in yeast extract buffer (YEB; 50 mM HEPES-KOH, pH 7.1, 100 mM β-glycerol phosphate, 50 mM NaF, 5 mM EGTA, 5 mM EDTA, 10% glycerol, 0.25% Tween 20, and 150 mM KCl). The pellet was resuspended and transferred to a 50 mL conical tube, pelleted again, resuspended 1:1 (*w/v*) in YEB containing protease inhibitors (cocktail tablet; Roche Diagnostics), 2 mM dithiothreitol, and 2 mM phenylmethylsulfonyl fluoride, and frozen dropwise by transfer pipet into liquid nitrogen. Cell pellets were then transferred to pre-chilled plastic tubes, and a cooled magnetic rod was added to each tube. Tubes were placed in a 6970EFM Freezer Mill (SPEX), pre-cooled in liquid nitrogen for 15 min, and cells were disrupted according to manufacturer’s instructions and the resulting powder was collected in a pre-chilled 50 mL conical tube in liquid nitrogen. The powder was thawed at 4 °C, collected in 1.5 mL microfuge tubes, and centrifuged at 20,000× *g* for 20 min at 4 °C. The supernatants (S1) were pooled and centrifuged again as described above. Supernatants (S2) were pooled after removal of the lipid layer at the surface. Protein concentrations were determined using a BCA assay (Sigma-Aldrich) and then normalized by dilution in YEB + protease inhibitors. 50 µL of this final dilution was saved as the load, and 5 µL of either 12CA5 or 9E10 antibody was added to 200 µL of the samples in a 2 mL microfuge tube. Tubes were incubated overnight at 4 °C. Next, a 50 µL 1:1 slurry of protein G Sepharose beads (GE Healthcare, Chicago, IL, USA) and YEB was added to each tube. Samples were incubated with beads for 2 h at 4 °C. Beads were pelleted at 10,000× *g* for 2 min at 4 °C and 40 µL of supernatant was saved as an unbound fraction. Beads were washed four times with 1 mL of YEB and the resulting beads were combined with sample buffer and analyzed by SDS-PAGE and Western blotting as described [21].

### 3.4. Immune-Complex In Vitro Kinase Assay

Kinase assays were performed essentially as described [41]. TOR-containing protein complexes were immuno-purified from yeast strains carrying plasmids expressing TOR chimeras using the 12CA5 antibody as described above. Next, 56 µL of kinase buffer (YEB + protease inhibitors, 2 mM dithiothreitol, 4 mM MnCl_2_, and 3 µg of recombinant GST-Ypk2), was added to beads that contained immuno-purified TOR complexes. Reactions were initiated by adding 4 µL of ATP mix (2 mM ATP, 5 µCi/µL [γ-^32^P] ATP, 3000 Ci/mmol, PerkinElmer, Waltham, MA, USA). Samples were incubated at 30 °C for 30 min and reactions were stopped by addition of 15 µL of 5X sample buffer and loaded onto two 7.5% SDS-PAGE gels. Proteins were transferred from one gel to nitrocellulose and probed with 12CA5 antibody to visualize TOR. The second gel was stained with Coomassie, exposed to a phosphorimager screen, and analyzed using a STORM 860 system (GE Healthcare, Chicago, IL, USA) and analyzed using software provided by the manufacturer and plotted using Excel (Microsoft, Redmond, WA, USA).

### 3.5. Actin Staining and Fluorescence Microscopy

Rhodamine–phalloidin staining of polarized actin was performed as described previously [51], with the following modifications. Strains were grown in SCD minus uracil and leucine media to early log phase and shifted to the non-permissive temperature of 37 °C for 2 h. Cells were then fixed, stained, and visualized as described [51]. For quantification of cells with depolarized actin cytoskeleton, a minimum of 100 small-budded or midsize-budded cells were counted for each condition. Cells were considered as having polarized actin if actin patches were concentrated in the bud and five or fewer patches were found in the mother cell. Cells were considered as partially polarized if actin patches were concentrated in the bud and there were more than 5 patches in the mother cell. Cells were considered as depolarized if patches were evenly distributed in both the bud and the mother cell.

### 3.6. Molecular Modeling

Molecular graphics and analyses were performed with the UCSF Chimera package. Chimera was developed by the Resource for Biocomputing, Visualization, and Informatics at the University of California, San Francisco (supported by NIGMS P41-GM103311).

## Figures and Tables

**Table 1:** Growth properties of *TOR1*–*TOR2* chimeras ^1^.

Plasmid	WT 30 °C + Rap	*tor1*Δ 30 °C + Rap	*tor2*Δ 30 °C	*tor2*Δ 37 °C	*tor2*Δ 37 °C + Sorbitol	*tor2*Δ 37 °C + YPD	*tor2*Δ 37 °C + YPD +Sorbitol
pRS315	0	0	0	ND	ND	ND	ND
pPL130	+++++	+++++	0	ND	ND	ND	ND
pPL132 ^2^	0	0	0	ND	ND	ND	ND
pPL089 ^2^	0	0	+++++	+++++	+++++	+++++	+++++
pPL268	+++++	+++++	+++++	+	++	+	+++++
pPL172 ^2^	ND	ND	+++++	+++++	+++++	+++++	+++++
pPL271	+++++	+++++	+++++	0	++	++	+++++
pPL273	+++++	+++++	+++++	+	++	++++	+++++
pPL175	+++++	+++++	+++++	++	+++	++++	+++++
pPL176	+++++	+++++	++++	+	+	++	+++++
pPL212	++++	+++++	+++++	+	++	++++	+++++
pPL180	+++++	+++++	++++	0	0	0	+
pPL184	+++	++++	++++	0	<+	<+	++++
pPL185	+++++	+++++	+++	0	0	0	<+
pPL209	+++++	+++++	+++	0	0	0	++++
pPL214	++++	++++	+++++	0	+	0	++++
pPL321 ^2^	ND	ND	+++++	+++++	+++++	+++++	+++++
pPL333 ^2^	ND	ND	+++++	+++	++++	++++	+++++
pPL270	+++++	+++++	0	ND	ND	ND	ND
pPL173	+++++	+++++	0	ND	ND	ND	ND
pPL177	++++	+++	0	ND	ND	ND	ND
pPL183	+++	++++	0	ND	ND	ND	ND
pPL182	++++	++++	0	ND	ND	ND	ND
pPL186	+++++	+++++	0	ND	ND	ND	ND

^1^ Cell growth was determined by relative colony size following incubation for 2–4 days on solid medium: +++++ corresponds to wild type (WT) growth and 0 corresponds to no growth. ND = Not Determined. + Rap = growth in the presence of 0.2 µg/mL rapamycin. The WT control strain refers to strain W303a and *tor1*Δ and *tor2*Δ strains are derived from this strain. Plasmid pRS315 is an empty control vector; pPL130 carries the *TOR1-1* gene; pPL132 carries the WT *TOR1* gene; pPL089 carries the wild type *TOR2* gene. Other plasmids are described in Figure 1 and Table 4. ^2^ Plasmid does not carry the *TOR1-1* allele. Therefore, its ability to confer TORC1 activity was tested by the ability to confer WT growth within a W303a *tor1*Δ strain at 37 °C.

**Table 2:** Predicted atomic contacts between Tor2-specific sequences and indicated TORC2 components within different chimeras ^1^.

	Chimera 172	Chimera 273	Chimera 212	Chimera 185	Chimera 333
Tor2: Intramolecular	28,174	8877	9107	9667	32,603
Tor2: Intermolecular	193	193	193	0	206
Lst8	0	0	0	0	140
Avo1	0	0	0	0	143
Avo2	86	65	0	21	21
Avo3	69	55	48	0	36
Observed Stable Complex	TORC2	TORC2	TORC1	TORC1	TORC1/2

^1^ Contacts listed were identified using the published structure of yeast TORC2 [30] and default “find clashes/contacts” settings in UCSF Chimera [42]. Specific regions of Tor2 that correspond to each chimera were used for this analysis, where interactions between each region and all other atoms within the structure were identified. Values listed refer to total number of predicted atomic contacts involving the Tor2-specific portion of each chimera.

**Table 3:** Yeast strains used in this study.

Strain	Genotype	Source
PLY061	W303a (*leu2-3,-112; his3-11,-15; trp1-1; ura3-1; ade2-1; can1-100; ssd1-d*)	[47]
PLY314	W303a/α *tor2::HIS/TOR2*	[48]
PLY497	W303a *tor1::TRP*	This study
PLY577	W303a *AVO1-13MYC:TRP1*	This study
PLY671	W303a *KOG1-13MYC:TRP1*	This study
PLY699	W303a *tor2::HIS3* [pPL089]	This study
PLY718	W303a *AVO3-13MYC:TRP1*	This study
PLY737	W303a *tor2::HIS3* [pPL273]	This study
PLY738	W303α *tor2::HIS3* [pPL273]	This study
PLY820	W303α *tor2::HIS3* [pPL172]	This study
PLY862	W303α *tor2::HIS3* [pPL176]	This study
PLY1020	W303a *tor2::HIS3* [pPL212]	This study
PLY1029	W303α *tor2::His3* [pPL185]	This study
PLY1164	W303a *BIT61-13MYC:TRP1*	This study
PLY1283	W303a *tor2::HIS3* [pPL321]	This study
PLY1285	W303a *tor2::HIS3* [pPL333]	This study
PLY1416	W303a *TCO89-13MYC:TRP1 TOR1::HIS3*	This study
PLY1417	W303a *BIT61-13MYC:TRP1 TOR2::HIS3* [pPL172]	This study
PLY1418	W303a *BIT61-13MYC:TRP1 TOR2::HIS3* [pPL273]	This study

**Table 4:** Plasmids used in this study.

Plasmid	Description	Source
pRS315	LEU2 CEN/ARS	[50]
pPL089	LEU2 CEN/ARS *TOR2*	[17]
pPL130	LEU2 CEN/ARS *TOR1-1*	[48]
pPL132	LEU2 CEN/ARS *TOR1*	[48]
pPL172	pPL132, Tor2 114-1770	This study
pPL173	pPL130 Tor2 428-681	This study
pPL175	pPL130, Tor2 590-946	This study
pPL176	pPL130, Tor2 682-946	This study
pPL177	pPL130, Tor2 787-946	This study
pPL180	pPL130, Tor2 1019-1770	This study
pPL182	pPL130, Tor2 1019-1236	This study
pPL183	pPL130, Tor2 1019-1399	This study
pPL184	pPL130, Tor2 1019-1542	This study
pPL185	pPL130, Tor2 1237-1770	This study
pPL186	pPL130, Tor2 1543-1770	This study
pPL209	pPL130, Tor2 1400-1770	This study
pPL212	pPL130, Tor2 682-1236	This study
pPL214	pPL130, Tor2 428-946 + 1019-1542	This study
pPL268	pPL130, Tor2 N-946	This study
pPL270	pPL130, Tor2 N-424	This study
pPL271	pPL130, Tor2 114-946	This study
pPL273	pPL130, Tor2 428-946	This study
pPL321	pPL130, *TOR2*	This study
pPL333	pPL321, Tor2 N-424 + 949-C	This study
pYE352	*URA3* 2-micron *YPK2-D239A*	[38]

## Supplementary Materials

The following are available online at http://www.mdpi.com/2218-273X/8/2/36/s1, Table S1: Chimera Switch Points.
