# Supplementary material for: Redesigning TOR Kinase to Explore the Structural Basis for TORC1 and TORC2 Assembly
Source: Biomolecules. 2018 Jun 1;8(2):36. doi: 10.3390/biom8020036 (PMC6023025; doi:10.3390/biom8020036)
Supplement: Supplementary file 1 [file biomolecules-08-00036-s001.pdf]

Supplemental Table S1. Chimera Switch Points

Plasmid	Nucleotide Position	Nucleotide sequence	Amino Acid Position	Translation
pPL268				
5' switch				
TOR1	NA	NA	NA	NA
TOR2	3HA	3HA	3HA-N	N-term
Result				
3'switch				
TOR1	2812	atccaagcgattatgcatatttt	938	IQAIMHIF
TOR2	2836	attcaagctattatgcatatttt	946	IQAIMHIF
Result		attcaagctattatgcatatttt		IQAIMHIF
pPL172				
5' switch				
TOR1	301	gatctactagtatccttggaattg	101	DLLVSLEL
TOR2	340	actactttgacctcattagcaagg	114	TTLTSLAR
Result		gatctactagtctcattagcaagg		DLLVSLAR
3'switch				
TOR1	5290	tttgaagtaatatccatggttcag	1764	FEVISMVQ
TOR2	5308	tttgaagtcatttctatgctaaca	1770	FEVISMLT
Result		tttgaagtcatttccatggttcag		FEVISMVQ
pPL271				
5' switch				
TOR1	301	gatctactagtatccttggaattg	101	DLLVSLEL
TOR2	340	actactttgacctcattagcaagg	114	TTLTSLAR
Result		gatctactagtctcattagcaagg		DLLVSLAR
3'switch				
TOR1	2812	atccaagcgattatgcatatttt	938	IQAIMHIF
TOR2	2836	attcaagctattatgcatatttt	946	IQAIMHIF
Result		attcaagctattatgcatatttt		IQAIMHIF
pPL273				

5' switch				
TOR1	1252	attgcatatgaagtcgggcccgat	418	IAYEVGPD
TOR2	1282	gatattgcatttgaagttggttcg	428	IAFEVGSS
Result		attgcatatgaagttggttcgagc		IAYEVGSS
3'switch				
TOR1	2812	atccaagcgattatgcatattttt	938	IQAIMHIF
TOR2	2836	attcaagctattatgcatattttt	946	IQAIMHIF
Result		attcaagctattatgcatattttt		IQAIMHIF
pPL175				
5' switch				
TOR1	1738	agcagattttcgttggtggaattc	580	SRFSLVEF
TOR2	1768	catcaatattccttgacggagttt	590	HQYSLTEF
Result		agcagattttcgttgacggagttt		SRFSLTEF
3'switch				
TOR1	2812	atccaagcgattatgcatattttt	938	IQAIMHIF
TOR2	2836	attcaagctattatgcatattttt	946	IQAIMHIF
Result		attcaagctattatgcatattttt		IQAIMHIF
pPL176				
5' switch				
TOR1	2014	aatttgagactcttgtttactgca	672	NLRLLFTA
TOR2	2044	aatttacgcctacttttcatggcg	682	NLRLLFMA
Result		aatttgagactccttttcatggcg		NLRLLFMA
3'switch				
TOR1	2812	atccaagcgattatgcatattttt	938	IQAIMHIF
TOR2	2836	attcaagctattatgcatattttt	946	IQAIMHIF
Result		attcaagctattatgcatattttt		IQAIMHIF
pPL212				
5' switch				
TOR1	2014	aatttgagactcttgtttactgca	672	NLRLLFTA
TOR2	2044	aatttacgcctacttttcatggcg	682	NLRLLFMA
Result		aatttgagactccttttcatggcg		NLRLLFMA

3'switch				
TOR1	3688	caacaaagaactaaagaagattgg	1230	QQRTKEDW
TOR2	3706	caacagaagaccaaagaagattgg	1236	QQKTKEDW
Result		caacagaagaccaaagaagattgg		QQKTKEDW
pPL180				
5' switch				
TOR1	3025	gttgctaagctacaaataacgctt	1009	VAKLQITL
TOR2	3055	atcattaaactacaaatcacaatt	1019	IIKLQITI
Result		gttgctaagctacaaatcacaatt		VAKLQITI
3'switch				
TOR1	5290	tttgaagtaatatccatggttcag	1764	FEVISMVQ
TOR2	5308	tttgaagtcatttctatgctaaca	1770	FEVISMLT
Result		tttgaagtcatttccatggttcag		FEVISMVQ
pPL184				
5' switch				
TOR1	3025	gttgctaagctacaaataacgctt	1009	VAKLQITL
TOR2	3055	atcattaaactacaaatcacaatt	1019	IIKLQITI
Result		gttgctaagctacaaatcacaatt		VAKLQITI
3'switch				
TOR1	4606	aatagagcatatagcgttattgtt	1536	NRAYSVIV
TOR2	4624	aatagagcatataatgttgttgtt	1542	NRAYNVVV
Result		aatagagcatatagcgttattgtt		NRAYSVIV
pPL185				
5' switch				
TOR1	3685	caacaaagaactaaagaagattgg	1229	QQRTKEDW
TOR2	3709	caacagaagaccaaagaagattgg	1237	QQKTKEDW
Result		caacaaagaactaaagaagattgg		QQRTKEDW
3'switch				
TOR1	5290	tttgaagtaatatccatggttcag	1764	FEVISMVQ
TOR2	5308	tttgaagtcatttctatgctaaca	1770	FEVISMLT
Result		tttgaagtcatttccatggttcag		FEVISMVQ

pPL209				
5' switch				
TOR1	4174	cattcacttcaattaaaggagaca	1392	HSLQLKET
TOR2	4198	aatgaattgcagctgaaggaaact	1400	NELQLKET
Result		cattcacttcaactgaaggaaact		HSLQLKET
3'switch				
TOR1	5290	tttgaagtaatatccatggttcag	1764	FEVISMVQ
TOR2	5308	tttgaagtcatttctatgctaaca	1770	FEVISMLT
Result		tttgaagtcatttccatggttcag		FEVISMVQ
pPL214		Switch points are the same as for 273 and 184 together.		
pPL321		Entirely *TOR2* sequence (with endogenous Promoter and Terminator) in pPL130 backbone		
pPL333				
5' switch				
TOR1	1246	attggtgatattgcatatgaagtc	416	IGDIAYEV
TOR2	1270	ataggtgatattgcatttgaagtt	424	IGDIAFEV
Result		ataggtgatattgcatatgaagtc		IGDIAYEV
3'switch				
TOR1	2815	gcgattatgcatatttttcaaacc	939	AIMHIFQT
TOR2	2845	gctattatgcatatttttcaaaac	949	AIMHIFQN
Result		gcgattatgcatatttttcaaaac		AIMHIFQN
pPL270				
5' switch				
TOR1	NA	NA	NA	NA
TOR2	3HA	3HA	3HA-N	N-term
Result				
3'switch				
TOR1	1246	attgcatatgaagtcgggcccgat	416	IAYEVGPD
TOR2	1270	attgcatttgaagttggttcgagc	424	IAFEVGSS
Result		attgcatatgaagttggttcgagc		IAYEVGSS
pPL173				

5' switch				
TOR1	1252	attgcatatgaagtcgggcccgat	418	IAYEVGPD
TOR2	1282	gatattgcatttgaagttggttcg	428	IAFEVGSS
Result		attgcatatgaagttggttcgagc		IAYEVGSS
3'switch				
TOR1	2017	aatttgagactcttgtttactgca	673	NLRLLFTA
TOR2	2041	aatttacgcctacttttcatggcg	N-681	NLRLLFMA
Result		aatttacgcctattgtttactgca		NLRLLFTA
pPL177				
5' switch				
TOR1	2329	actataggtgagctatctgttgta	777	TIGELSVV
TOR2	2359	gttttgggtgaactatctgttgtt	787	VLGELSVV
Result		actataggtgagctatctgttgtt		TIGELSVV
3'switch				
TOR1	2812	atccaagcgattatgcatattttt	938	IQAIMHIF
TOR2	2836	attcaagctattatgcatattttt	946	IQAIMHIF
Result		attcaagctattatgcatattttt		IQAIMHIF
pPL183				
5' switch				
TOR1	3025	gttgctaagctacaaataacgctt	1009	VAKLQITL
TOR2	3055	atcattaaactacaaatcacaatt	1019	IIKLQITI
Result		gttgctaagctacaaatcacaatt		VAKLQITI
3'switch				
TOR1	4177	cattcacttcaattaaaggagaca	1393	HSLQLKET
TOR2	4195	aatgaattgcagctgaaggaaact	1399	NELQLKET
Result		aatgaattgcagctgaaggaaact		NELQLKET
pPL182				
5' switch				
TOR1	3025	gttgctaagctacaaataacgctt	1009	VAKLQITL
TOR2	3055	atcattaaactacaaatcacaatt	1019	IIKLQITI
Result		gttgctaagctacaaatcacaatt		VAKLQITI

3'switch				
TOR1	3688	caacaaagaactaaagaagattgg	1230	QQRTKEDW
TOR2	3706	caacagaagaccaaagaagattgg	1236	QQKTKEDW
Result		caacagaagaccaaagaagattgg		QQKTKEDW
pPL186				
5' switch				
TOR1	4603	aatagagcatatagcgttattgtt	1535	NRAYSVIV
TOR2	4627	aatagagcatataatgttgttgtt	1543	NRAYNVVV
Result		aatagagcatataatgttgttgtt		NRAYNVVV
3'switch				
TOR1	5290	tttgaagtaatatccatggttcag	1764	FEVISMVQ
TOR2	5308	tttgaagtcatttctatgctaaca	1770	FEVISMLT
Result		tttgaagtcatttccatggttcag		FEVISMVQ
